# Editorial: Diverticulitis - A neglected disease despite its clinical burden

**DOI:** 10.3389/fmed.2025.1748075

**Published:** 2025-12-04

**Authors:** Antonio Tursi, Savvas Papagrigoriadis, Wolfgang Kruis

**Affiliations:** 1 Territorial Gastroenterology Service, ASL BAT, Andria, BT, Italy; 2 Department of Medical and Surgical Sciences, School of Medicine, Catholic University, Rome, Italy; 3 Department of Colorectal Surgery, King's College Hospital, London, United Kingdom; 4 IASO General Hospital, Athens, Greece; 5 Faculty of Medicine, University of Cologne, Cologne, Germany

**Keywords:** diverticulitis, SUDD, epidemiology, pathogenesis, management

Diverticulosis of the colon and its clinical manifestation, Diverticular Disease (DD), are highly frequent conditions. The related symptoms and complications are a burden to patients and a challenge to healthcare systems. Diverticulitis ranks among the ten most frequent GI diagnoses in the United States, with an annual incidence of approximately 180 per 100,000 individuals, resulting in approximately 200,000 hospital admissions per year (sixth most common reason for hospitalization) and an estimated healthcare expenditure of more than $6.3 billion/year (1). The incidence and prevalence of DD are both increasing worldwide, and in-hospital mortality approaches 7% (2).

Apparently, the discourse on DD underscores the pressing need for advanced management strategies and new treatments. Although research in this field is ongoing, more basic and clinical scientific work is urgently needed. The pathogenesis and pathophysiology of DD are not completely understood. Genetic predisposition and lifestyle, particularly in a Western environment, are considered key factors. However, important questions remain unanswered, such as the impact of low-grade inflammation and the factors leading from asymptomatic diverticulosis to symptomatic uncomplicated DD (SUDD) and acute diverticulitis (AD) (3).

This Research Topic collects several studies that cover different aspects of DD.

Two studies were conducted in the setting of SUDD. The first study, conducted in Japan, focused its attention on the potential role of bile acids (BAs) in the pathogenesis of SUDD. The authors found that fecal BA concentrations were significantly increased in patients with SUDD compared with controls, suggesting that fecal BAs may be involved in the pathogenesis of SUDD, and that controlling fecal BA levels could be a therapeutic approach for SUDD (Jono et al.). The second study, conducted in Italy, examined the role of a post-biotic, sodium butyrate, in modulating gut microbiota (GM) expression and managing SUDD symptoms. Using a microencapsulated, colonic-release formulation, the authors found that sodium butyrate reduces proinflammatory bacterial taxa and improves abdominal pain (Tursi et al.).

Two studies from Greece investigated the AD setting. In the first, the author discussed the current and evolving surgical treatments for this complex disease (Papagrigoriadis and Charalampopoulos); in the second, the same author discussed the treatment of fistulating diverticulitis. By analyzing histopathology and epidemiological characteristics, the author proposed an interesting point of view on the need to consider this DD phenotype as a distinct clinical entity rather than a simple complication of the disease (Papagrigoriadis et al.).

In an interesting case report from Romania, the authors reported an unusual case of jejunal diverticulitis (Chiorescu et al.).

These studies explore new approaches to the pathogenesis and treatment of this neglected condition. Regarding the role of BAs, we know that increased levels of fecal BAs, especially secondary BAs, have been reported to induce intestinal inflammation and diarrhea (4). A study by Jono et al. revealed interesting findings: 1. There is no enhanced production of BAs in SUDD, but high levels are detected in the feces of patients with SUDD; and 2. calprotectin levels are also detected and are linked to fecal BA levels. This could be due to an abnormality in the ileal BA transporter that causes a decrease in BA absorption and an increase in BA levels in the colon. Therefore, this study suggests that BA malabsorption may cause low-grade inflammation and be a contributing factor to GM imbalance and symptom occurrence in these patients. In this way, a second study conducted with the same patient population opened up new therapeutic prospects. We know that butyrate, a short-chain fatty acid (SCFA), is an important energy source for colonocytes, regulates motility, pH, and blood flow, improves mucosal barriers, and exerts significant anti-inflammatory and antimicrobial properties (5). The study from Tursi et al. revealed two interesting findings: 1. Butyrate is more effective in patients with more severe GM imbalance, significantly improving its alpha- and beta-diversity; and 2. abdominal pain improvement is directly linked to the efficacy of butyrate. In other words, SUDD patients with more severe abdominal pain have higher GM imbalance and respond better to butyrate supplementation. This means that, by enhancing the metabolism of colonocytes with butyrate supplementation, we could influence not only the restoration of a healthier GM, but also modulate the other factors involved in the pathophysiology of the disease (such as visceral hypersensitivity and low-grade inflammation) (3).

The new information about the pathogenesis of the disease could also influence its surgical management. This means that not only are the surgical techniques evolving toward less invasive and more patient-focused techniques (Papagrigoriadis and Charalampopoulos), but also that specific disease phenotypes, such as fistulating diverticulitis, must be revised according to the new evidence. For example, we know that Crohn's disease (CD) and diverticulitis of the colon share several characteristics (e.g., the tendency toward fibrosis, the expression of certain pro-fibrotic cytokines) (6). Fistulating diverticulitis also appears to have pathological features very similar to those of CD (e.g., granulomatous inflammation, granulomatous vasculitis with mural lymphoid aggregates, non-necrotizing granulomatous inflammation situated at the outer edge of the muscularis propria, and active vasculitis with transmural lymphoplasmacytic inflammation) (7, 8). This suggests that surgical techniques currently adopted for CD (9) could soon be applied to this phenotype of diverticulitis. Unfortunately, a surgical approach remains the main therapeutic approach to jejunal diverticulitis due to its rare occurrence and its non-specific symptoms (Chiorescu et al.).

In summary, this Research Topic shows that there is significant progress in understanding the causes of this disease and managing these patients. In view of the epidemiological importance of this condition, further clinical research is needed to further refine patient care.
